# Analysis and application of a machine learning-based assisted diagnosis method for myofascial pelvic pain syndrome

**DOI:** 10.3389/fmed.2026.1627891

**Published:** 2026-04-14

**Authors:** Hang Yu, Tingwei Xiao, Yingying Li, Zhuyin Li, Jiaxi Liu, Hongguo Zhao, Yanhua Dong, Dongxia Liu, Lei Li, Xiaohua Luo

**Affiliations:** The Department of Obstetrics and Gynecology, The Third Affiliated Hospital of Zhengzhou University, Zhengzhou, China

**Keywords:** assisted diagnosis method, graphical user interface, machine learning, myofascial pelvic pain syndrome, prediction model

## Abstract

**Objectives:**

The research aims to leverage machine learning techniques to better understand the diagnosis of myofascial pelvic pain syndrome (MPPS) and to develop useful tools for clinical practice.

**Methods:**

This study retrospectively analyzed clinical data from female patients. Between January 2021 and December 2024, 1,204 MPPS cases and 1,217 healthy women from the Pelvic Floor Rehabilitation Center of Zhengzhou University’s Third Affiliated Hospital were enrolled. After screening, 1,136 MPPS patients and 1,136 healthy controls were selected. Using *Python 3.9*, we developed prediction models with 10 machine learning algorithms: logistic regression, support vector machine (SVM), decision tree (DT), random forest (RF), eXtreme gradient boosting (XGBoost), light gradient boosting machine (LightGBM), adaptive boosting (AdaBoost), categorical boosting (CatBoost), k-nearest neighbors (KNN), and backpropagation (BP). Five-fold cross-validation was used to prevent overfitting. The models’ performance was evaluated using accuracy, precision, recall, F1 score, and the area under the receiver operating characteristic curve (AUC-ROC) to assess each algorithm’s diagnostic value for MPPS.

**Results:**

The top four models in terms of AUC, ranked from highest to lowest, were RF, CatBoost, XGBoost, and LightGBM. The top four models in terms of accuracy, ranked from highest to lowest, were CatBoost, RF, XGBoost, and LightGBM. Moreover, the top four models in terms of area under the decision curve (AUDC), ranked from highest to lowest, were CatBoost, LightGBM, XGBoost, and RF. Furthermore, we created a web-based graphical user interface (GUI) for MPPS prediction. It can be packaged for cross-platform use, thereby streamlining diagnosis and improving accessibility for healthcare providers.

**Conclusion:**

In conclusion, this study compared 10 machine learning algorithms for diagnosing myofascial pelvic pain syndrome. The CatBoost model showed superior performance in terms of accuracy and clinical utility. In addition, a cross-platform web-based GUI was developed, streamlining diagnosis for healthcare providers and potentially improving patient outcomes.

## 1 Introduction

Myofascial pelvic pain syndrome (MPPS) is a complex and common chronic disease in women that has a serious impact on patients’ quality of life (1). Its pain symptoms are diverse, not only manifesting as pain in the pelvic region but also accompanied by problems such as muscle tension and functional disorders (2). Since its pathogenesis involves interactions among multiple systems, such as muscles, fascia, and nerves, and the symptoms lack specificity, the diagnosis of this condition can be challenging. Traditional diagnostic methods primarily rely on the doctor’s experience, physical examinations, and limited imaging studies. These methods often make it difficult to accurately identify the causes and pathology of the disease, leading to misdiagnosis and missed diagnosis and negatively affecting treatment outcomes (3).

With the continuous development of machine learning technology in the medical field, its powerful data analysis capabilities have brought new hope for the diagnosis of MPPS. Machine learning algorithms can handle large volumes of complex medical data effectively (4–6). By uncovering the hidden patterns and relationships within the data, these algorithms can provide more accurate support for disease diagnosis (7). Among the numerous machine learning algorithms, logistic regression can build a prediction model based on multiple characteristic variables, effectively analyze the association between diseases and various factors, and provide a feasible approach for disease diagnosis (8). Support vector machine (SVM) is proficient in handling high-dimensional data, extracting hidden patterns, and assisting doctors in identifying relevant features for disease diagnosis (9). The decision tree (DT) algorithm classifies data using an intuitive tree structure that clearly presents the relationship between features and diseases, making it convenient for clinical applications (10). The random forest (RF) method integrates the results of multiple decision trees, enhancing the model’s stability and generalization abilities and making the classification prediction more accurate and reliable (11). Boosting algorithms such as eXtreme gradient boosting (XGBoost), light gradient boosting machine (LightGBM), adaptive boosting (AdaBoost), and categorical boosting (CatBoost) can handle data noise and non-linear relationships, thereby improving the accuracy of disease diagnosis (12). The k-nearest neighbors (KNN) algorithm classifies data based on the distance between data points and predicts disease state using data from similar patients (13). The backpropagation (BP) algorithm optimizes models within neural networks, uncovers the deep features of data, and provides accurate support for disease diagnosis (14). These models are among the most commonly used machine learning algorithms, and there may be significant differences in their performance across different problems, particularly in disease prediction. Therefore, it is essential to compare the performance of these models in predicting MPPS in order to establish a more accurate prediction model for MPPS.

This study aimed to evaluate the performance of 10 machine learning methods, namely logistic regression, SVM, DT, RF, XGBoost, LightGBM, AdaBoost, CatBoost, KNN, and BP, in predicting the diagnosis of MPPS. By collecting extensive data from various sources, including detailed clinical baseline data such as height, weight, pregnancy and childbirth history, symptoms, signs, pelvic floor pressure assessments, and modified Oxford muscle strength assessments, appropriate machine learning algorithms were selected and optimized to establish a reliable auxiliary diagnostic model for MPPS. At the same time, the performance of the model was comprehensively evaluated and compared with traditional diagnostic methods. Finally, the model was applied in clinical practice to verify its effectiveness and practicality in assisting doctors with diagnosis, thereby providing more powerful support for the diagnosis and treatment of myofascial pelvic pain syndrome.

## 2 Objectives and methods

### 2.1 Objectives

This research represents a retrospective analysis of clinical data obtained from female patients who underwent pelvic floor evaluations for chronic pelvic pain at the Pelvic Floor Rehabilitation Center of the Third Affiliated Hospital of Zhengzhou University between January 2021 and December 2024. A total of 1,204 cases of MPPS were analyzed, alongside 1,217 healthy women who visited the hospital for routine physical examinations. After applying inclusion and exclusion criteria, 1,136 patients were categorized into the MPPS group, while 1,136 healthy women were assigned to the non-MPPS group for this analysis. The diagnosis of MPPS was established in accordance with the gold standard recommended by the European Association of Urology (EAU) guidelines (15).

### 2.2 Inclusion and exclusion criteria

#### 2.2.1 Inclusion criteria

The inclusion criteria were as follows:

(a) Diagnosis of MPPS.
(b) Complete clinical data.
(c) No disease-related therapy within 1 month prior to participation.
(d) Age 18 years or older, and a history of sexual activity.

#### 2.2.2 Exclusion criteria

The exclusion criteria were as follows:

(a) Inadequate or incomplete clinical data.
(b) Presence of other pelvic floor disorders, such as pelvic organ prolapse or urinary incontinence.
(c) Chronic pelvic pain attributed to specific known causes, including (but not limited to) pelvic venous congestion syndrome, adenomyosis, endometriosis, and uterine fibroids (16).

### 2.3 Data collection

Table 1 shows that this study encompassed 23 clinical data features. Eight baseline clinical data elements were collected, namely age, height, weight, body mass index (BMI), gravidity, parity, hysterectomy status, and perineal laceration (17). Moreover, the dataset incorporated 11 pelvic floor pressure electromyography (EMG)-related clinical screening data points (18). In addition, the modified Oxford muscle strength grading was included in the study (19).

**Table 1:** Feature index of MPPS.

Feature index	Feature
1	Age (years)
2	Height (cm)
3	Weight (Kg)
4	BMI (Kg/m^2^)
5	Gravidity(times)
6	Parity(times)
7	Pre-resting phase average value(mmHg)
8	Pre-resting phase coefficient of variation
9	Rapid contraction phase maximum value(mmHg)
10	Rapid contraction phase maximum value relaxation time(s)
11	Tension contraction phase average value(mmHg)
12	Tension contraction phase coefficient of variation
13	Tension contraction phase relaxation time(s)
14	Endurance contraction phase average value(mmHg)
15	Endurance contraction phase coefficient of variation
16	Post-resting phase average value(mmHg)
17	Post-resting phase coefficient of variation
18	Deep layer type I muscle
19	Deep layer type II muscle
20	Superficial layer type I muscle
21	Superficial layer type II muscle
22	Hysterectomy status (Yes = 1, No = 0)
23	Perineal laceration (Yes = 1, No = 0)

### 2.4 Statistical analysis methods

This study conducted all data processing, feature engineering, and model construction using Python 3.9. The core libraries include Pandas 2.1.4, NumPy 1.26.2, Scikit-learn 1.3.2, XGBoost 2.0.2, LightGBM 4.1.0, CatBoost 1.2.2, and Matplotlib 3.8.2. A total of 10 machine learning algorithms were used for model building: logistic regression, SVM, DT, RF, XGBoost, LightGBM, AdaBoost, CatBoost, KNN, and BP neural network. A comprehensive analytical workflow (data preprocessing, feature processing, hyperparameter optimization, and cross-validation) was adopted to ensure the model’s reliability and generalization abilities.

#### 2.4.1 Data and feature preprocessing

The quality of the collected clinical data was assessed and optimized to minimize the impact of low-quality data on model performance. For missing values, we first calculated the missing rate of each feature: Features with >10% missing values were excluded, and the remaining missing values were filled with median (continuous features). Mode imputation was applied to categorical variables. Outliers were identified using the interquartile range (IQR; values outside Q1–1.5 × IQR or Q3 + 1.5 × IQR) and replaced with the median to mitigate their influence. All continuous clinical features (e.g., age, BMI, and pelvic floor pressure indicators) were normalized using Z-score standardization to map the data to a standard normal distribution (*μ* = 0, *σ* = 1), eliminating feature scale differences and enhancing model convergence and prediction accuracy. Categorical features (hysterectomy status and perineal laceration) were converted to binary variables (Yes = 1 and No = 0) for subsequent modeling. Given the limited number of features employed in this study, feature selection was not conducted to prevent the loss of critical data information.

#### 2.4.2 Model hyperparameter optimization

To reduce the influence of unreasonable hyperparameter settings on model performance, a Grid Search Cross-Validation (GridSearchCV) strategy was adopted for hyperparameter optimization across all 10 machine learning algorithms, using 5-fold cross-validation during the search process. For each algorithm, we identified the core hyperparameters that significantly influence model performance and established a reasonable search range (Table 2), and the accuracy score was used as the evaluation index for optimal parameter selection. The hyperparameter combination with the highest average 5-fold cross-validation accuracy on the training set was selected as the optimal hyperparameter of the corresponding model, which was used for the final model training and performance evaluation.

**Table 2:** Core hyperparameter search ranges for 10 machine learning algorithms.

Algorithm	Core hyperparameters	Search range
Logistic Regression	C, solver	[0.01, 0.1, 1, 10, 100]; [‘liblinear’, ‘saga’]
SVM	C, kernel, gamma	[0.01, 0.1, 1, 10]; [‘rbf’, ‘linear’]; [‘scale’, ‘auto’]
DT	max_depth, min_samples_split	[3, 5, 7, 9, None]; (2, 5, 10)
RF	n_estimators, max_depth	[100, 200, 300, 500]; [5, 10, 15, None]
XGBoost	n_estimators, learning_rate, max_depth	[100, 200, 300]; [0.01, 0.1, 0.2]; (3, 5, 7)
LightGBM	n_estimators, learning_rate, num_leaves	[100, 200, 300]; [0.01, 0.1, 0.2]; [31, 63, 127]
AdaBoost	n_estimators, learning_rate	[50, 100, 200]; [0.01, 0.1, 1]
CatBoost	iterations, learning_rate, depth	[100, 200, 300]; [0.01, 0.1, 0.2]; (3, 5, 7)
KNN	n_neighbors, weights	(3, 5, 7, 9); [‘uniform’, ‘distance’]
BP	hidden_layer_sizes, activation, solver	[(64,), (32, 64), (64, 32)]; [‘relu’, ‘sigmoid’]; [‘adam’, ‘sgd’]

#### 2.4.3 Model verification and performance evaluation

The processed dataset (1,136 MPPS patients and 1,136 healthy controls) was randomly divided into training and test sets at a 7:3 ratio (random state = 42) for model training, optimization, and independent evaluation. Five-fold cross-validation was performed on the training set to prevent overfitting and optimize model parameters, and the core hyperparameter search ranges for the machine learning algorithms are provided in Table 2.

The optimized models were evaluated on the test set using accuracy, precision, recall, F1 score, area under the receiver operating characteristic curve (AUC-ROC), and area under the decision curve (AUDC). All metrics were calculated from the confusion matrix, and the 95% confidence interval of the AUC was obtained using 1,000 bootstrap resamplings. Performance comparisons were conducted to assess the diagnostic value of each model for MPPS.

#### 2.4.4 Model verification and performance evaluation

To ensure model reliability and prevent data leakage, a two-stage validation strategy was adopted: The stratified dataset (2,272 samples: 1136 MPPS patients and 1,136 controls) was divided into 70% training and 30% independent test sets, with the test set strictly reserved for final evaluation. Five-fold cross-validation was applied to the training set for hyperparameter optimization and overfitting prevention, and the optimal model was evaluated using accuracy, precision, recall, F1 score, AUC (with a 95% confidence interval estimated through 1,000 bootstrap resamplings), and AUDC, ensuring objective and credible results.

**Figure 1:**
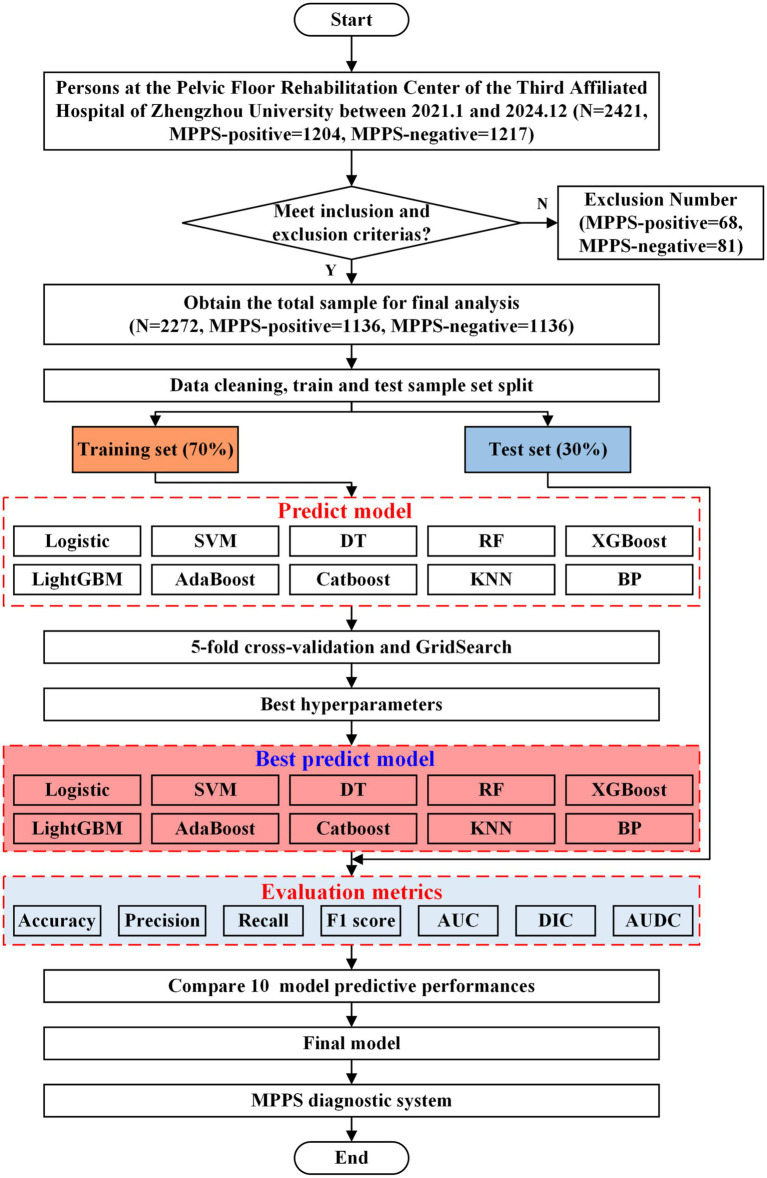
Modeling and analysis process.

The modeling and analysis process is shown in Figure 1.

## 3 Results

### 3.1 Feature importance analysis

**Figure 2:**
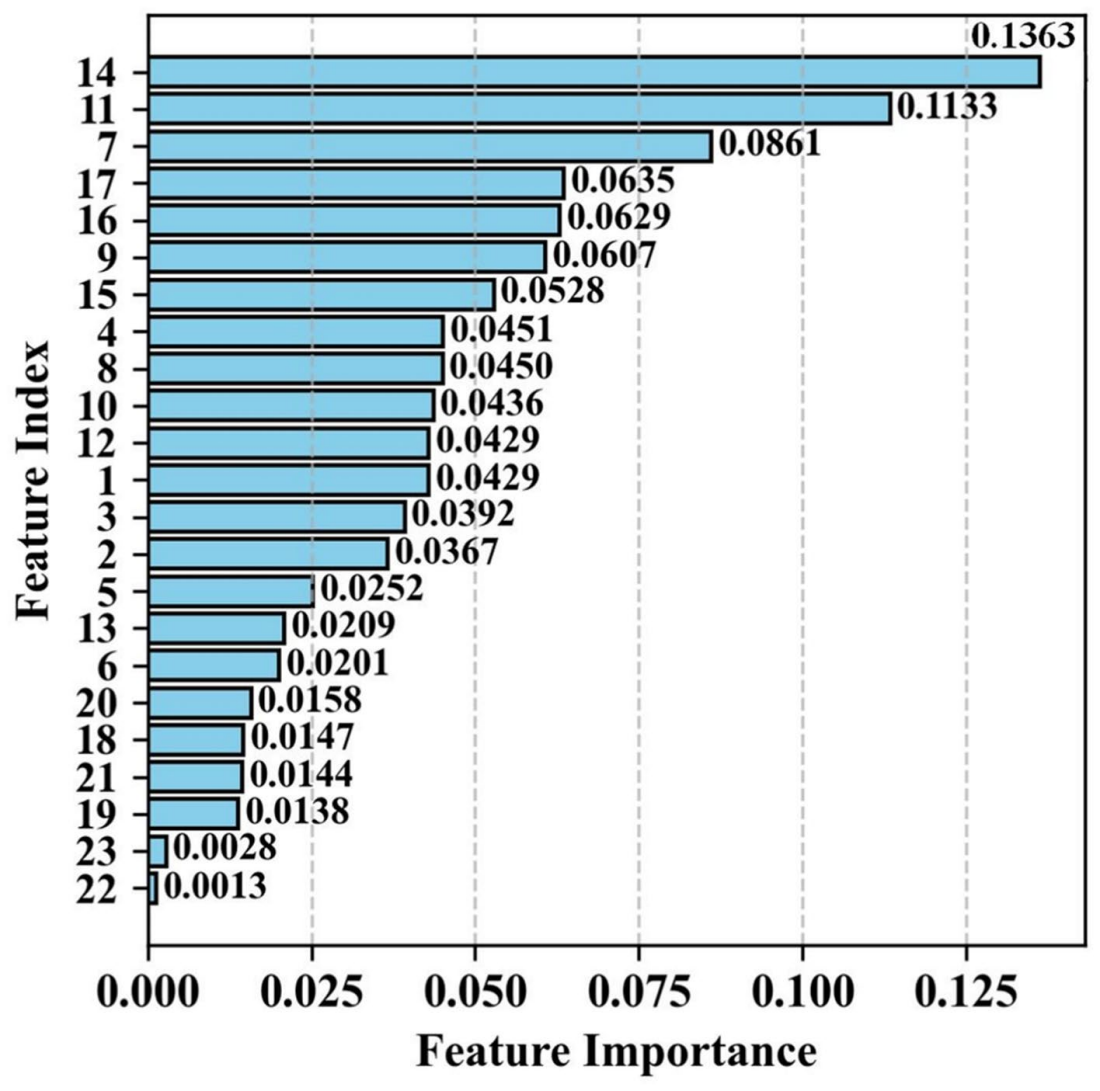
Feature importance analysis.

To further explore the model’s diagnostic logic and improve clinical interpretability, we analyzed the importance of features and quantified each feature’s contribution to MPPS diagnosis, as shown in Figure 2. The top five contributing features were endurance contraction phase average value, tension contraction phase average value, pre-resting phase average value, post-resting phase coefficient of variation, and post-resting phase average value, with importance weights of 0.1363, 0.1133, 0.0861, 0.0635, and 0.0629, respectively. Pelvic floor EMG-related features accounted for over 60% of the total importance, while baseline clinical features accounted for approximately 25%, suggesting that pelvic floor muscle function is the core diagnostic factor for MPPS, consistent with its clinical pathogenesis.

### 3.2 Predictive model performance analysis

**Figure 3:**
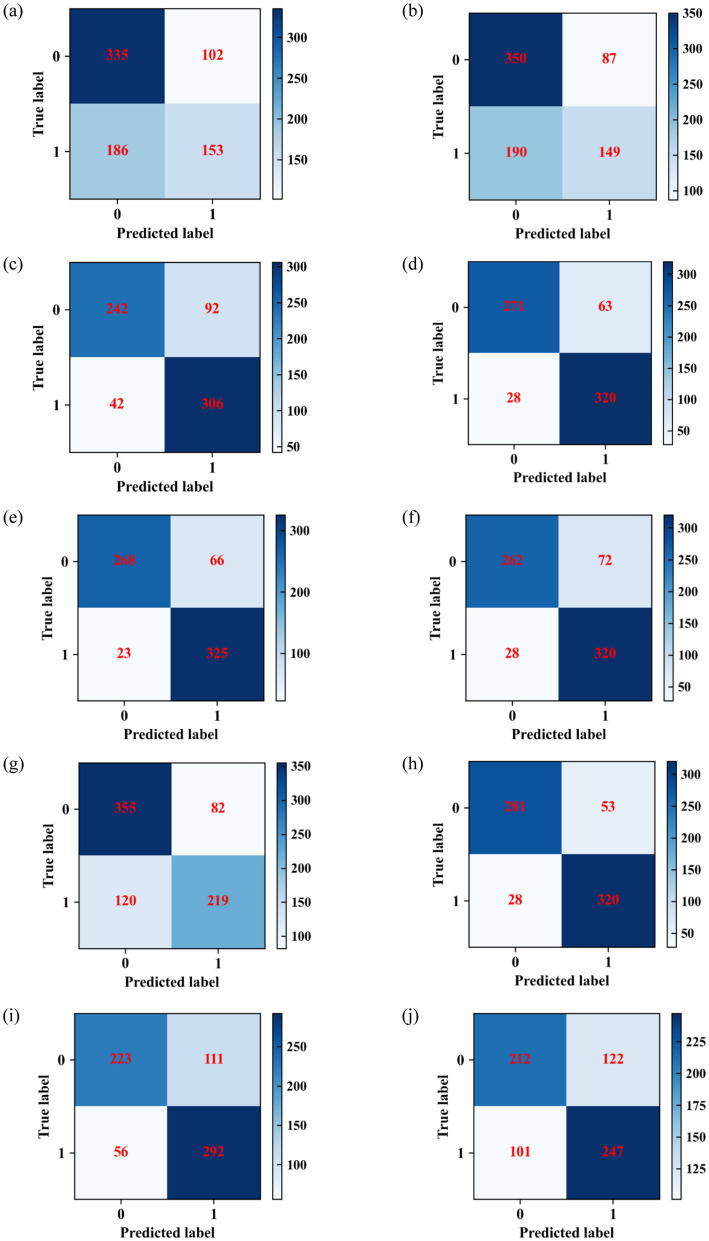
Confusion matrices of **(a)** logistic regression, **(b)** SVM, **(c)** DT, **(d)** RF, **(e)** XGBoost, **(f)** LightGBM, **(g)** AdaBoost, **(h)** CatBoost, **(i)** KNN, and **(j)** BP algorithms.

**Figure 4:**
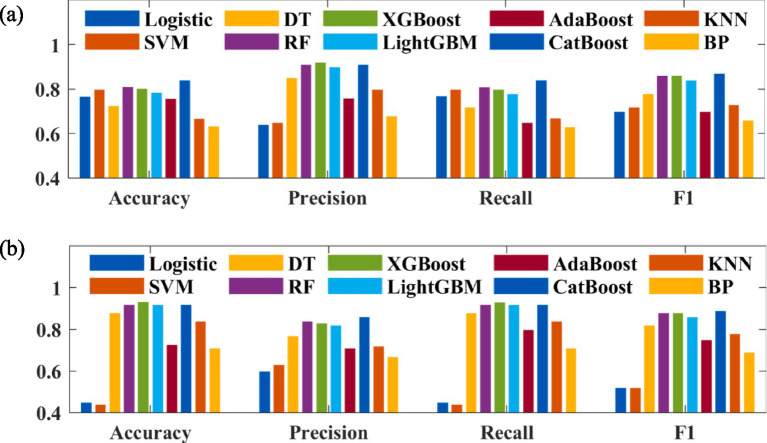
Evaluation indicators of **(a)** label 0 and **(b)** label 1.

The confusion matrices of the prediction models based on logistic regression, SVM, DT, RF, XGBoost, LightGBM, AdaBoost, CatBoost, KNN, and BP algorithms are shown in Figure 3. The evaluation indicators of these prediction models are presented in Figure 4. The accuracy values of the prediction models based on logistic regression, SVM, DT, RF, XGBoost, LightGBM, AdaBoost, CatBoost, KNN, and BP algorithms for predicting negative cases were 0.767, 0.8, 0.7246, 0.8114, 0.8024, 0.7844, 0.7579, 0.8413, 0.6677, and 0.6347, respectively; the precision values of these models were 0.64, 0.65, 0.85, 0.91, 0.92, 0.9, 0.76, 0.91, 0.8, and 0.68, respectively; the recall values of these models were 0.77, 0.8, 0.72, 0.81, 0.8, 0.78, 0.65, 0.84, 0.67, and 0.63, respectively; and the F1 scores of these model were 0.7, 0.72, 0.78, 0.86, 0.86, 0.84, 0.7, 0.87, 0.73, and 0.66, respectively. The accuracy values of the prediction models based on logistic regression, SVM, DT, RF, XGBoost, LightGBM, AdaBoost, CatBoost, KNN, and BP algorithms for predicting positive cases were 0.451, 0.44, 0.8793, 0.9195, 0.9339, 0.9195, 0.7273, 0.9195, 0.8391, and 0.7098, respectively; the precision values of these models were 0.6, 0.63, 0.77, 0.84, 0.83, 0.82, 0.71, 0.86, 0.72, and 0.67, respectively; the recall values of these models were 0.45, 0.44, 0.88, 0.92, 0.93, 0.92, 0.8, 0.92, 0.84, and 0.71, respectively; and the F1 scores of these models were 0.52, 0.52, 0.82, 0.88, 0.88, 0.86, 0.75, 0.89, 0.78, and 0.69, respectively.

**Figure 5:**
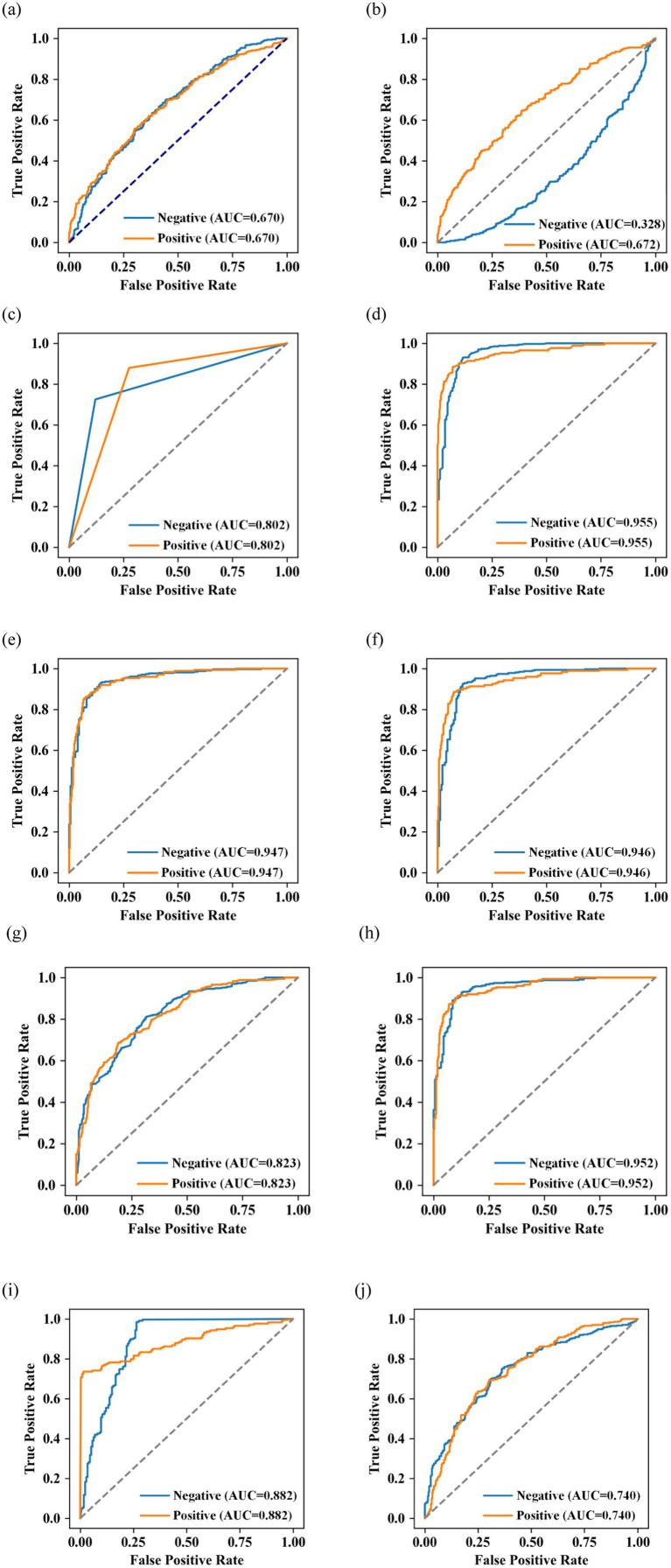
ROC curves of **(a)** logistic regression, **(b)** SVM, **(c)** DT, **(d)** RF, **(e)** XGBoost, **(f)** LightGBM, **(g)** AdaBoost, **(h)** CatBoost, **(i)** KNN, and **(j)** BP algorithms.

The ROC curves of the prediction models based on logistic regression, SVM, DT, RF, XGBoost, LightGBM, AdaBoost, CatBoost, KNN, and BP algorithms are shown in Figure 5. The AUC values of these models for predicting negative cases were 0.67 (95% confidence interval: [0.6333 0.7074]), 0.3283 (95% confidence interval: [0.2894 0.3641]), 0.8019 (95% confidence interval: [0.7703 0.8314]), 0.9548 (95% confidence interval: [0.9397 0.9692]), 0.9468 (95% confidence interval: [0.9299 0.9616]), 0.9458 (95% confidence interval: [0.9297 0.9623]), 0.836 (95% confidence interval: [0.805 0.8641]), 0.9515 (95% confidence interval: [0.9361 0.9652]), 0.8819 (95% confidence interval: [0.8548 0.9064]), and 0.7403 (95% confidence interval: [0.7013 0.7752]), respectively. The AUC values of these models for predicting positive cases were 0.67 (95% confidence interval: [0.6275 0.7066]), 0.6717 (95% confidence interval: [0.6366 0.7099]), 0.8019 (95% confidence interval: [0.7717 0.8302]), 0.9548 (95% confidence interval: [0.9388 0.9697]), 0.9468 (95% confidence interval: [0.9285 0.9624]), 0.9458 (95% confidence interval: [0.9277 0.9611]), 0.836 (95% confidence interval: [0.807 0.8618]), 0.9515 (95% confidence interval: [0.9354 0.9661]), 0.8819 (95% confidence interval: [0.8563 0.906]), and 0.7403 (95% confidence interval: [0.7056 0.7815]), respectively.

**Figure 6:**
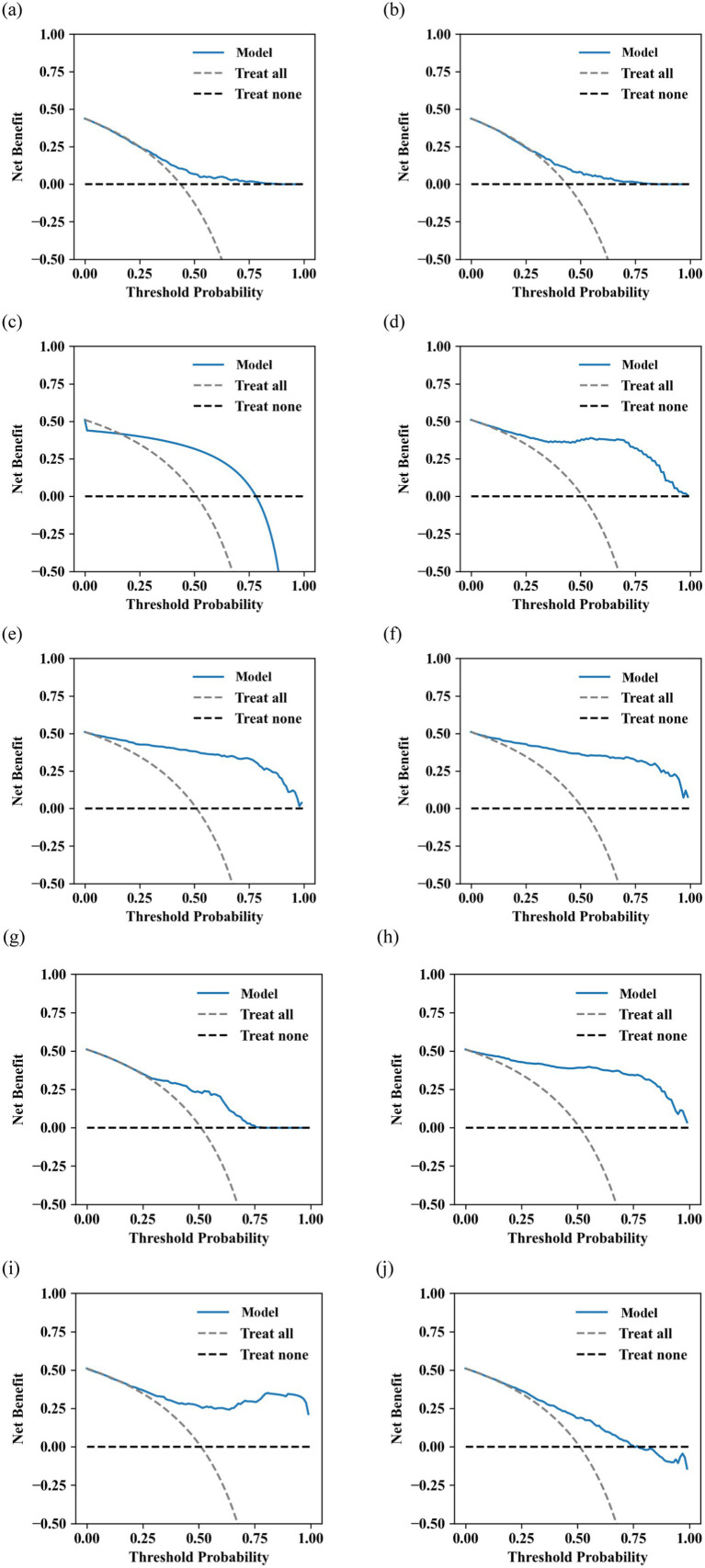
DCA of **(a)** logistic regression, **(b)** SVM, **(c)** DT, **(d)** RF, **(e)** XGBoost, **(f)** LightGBM, **(g)** AdaBoost, **(h)** CatBoost, **(i)** KNN, and **(j)** BP algorithms.

The decision curve analysis (DCA) of the prediction models based on logistic regression, SVM, DT, RF, XGBoost, LightGBM, AdaBoost, CatBoost, KNN, and BP algorithms is shown in Figure 6. The AUDC values of these models for predicting negative cases are 0.136, 0.1352, −0.199, 0.3389, 0.3569, 0.3593, 0.217, 0.3674, 0.3318, and 0.1932, respectively.

### 3.3 Model calibration analysis

**Figure 7:**
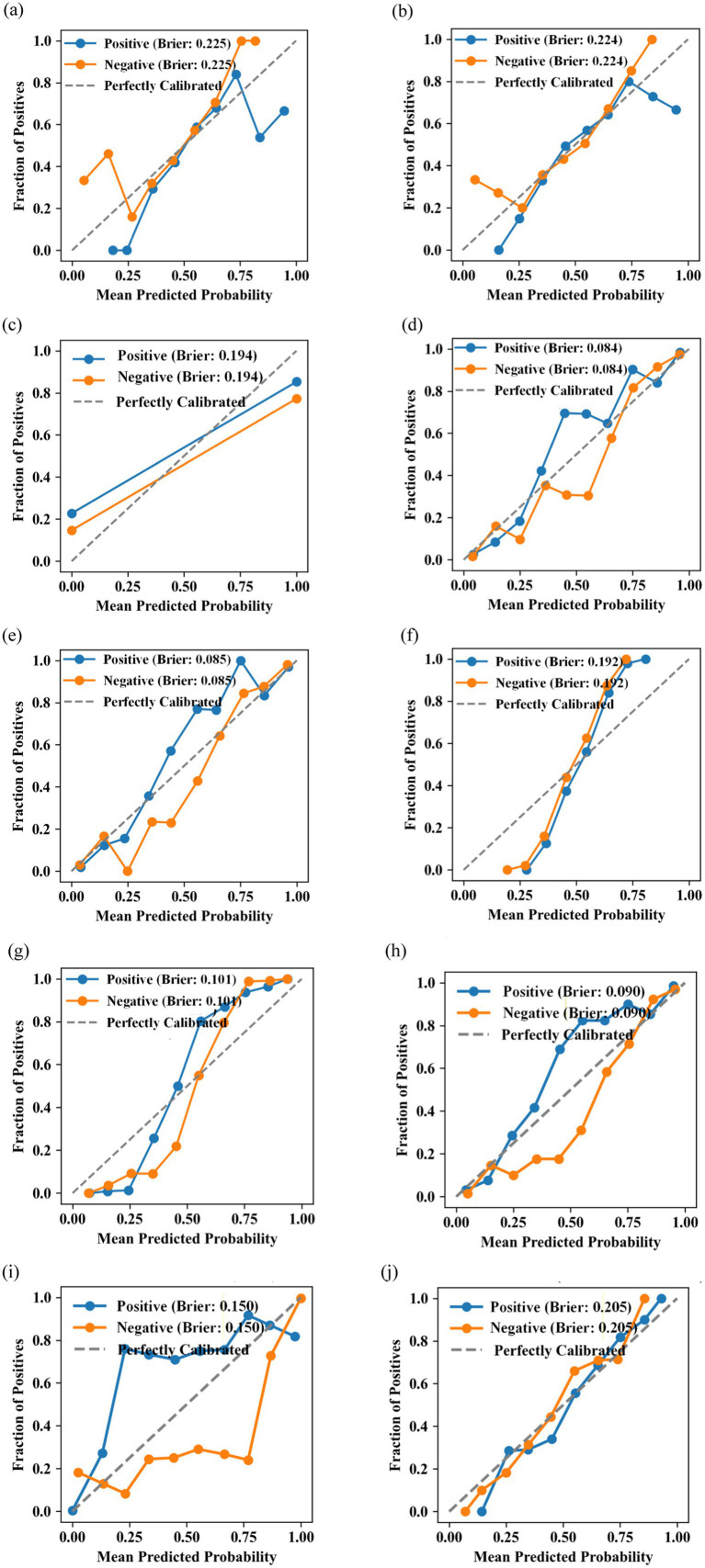
Calibration plots of **(a)** logistic regression, **(b)** SVM, **(c)** DT, **(d)** RF, **(e)** XGBoost, **(f)** LightGBM, **(g)** AdaBoost, **(h)** CatBoost, **(i)** KNN, and **(j)** BP algorithms.

We further analyzed the calibration of the models, which is shown in Figure 7. RF, XGBoost, AdaBoost, and CatBoost displayed low Brier scores, and their calibration curves were close to the diagonal reference line, demonstrating excellent calibration. In contrast, other models showed obvious deviations from the diagonal reference line, reflecting relatively poorer calibration.

### 3.4 Statistical comparison of AUC using the DeLong test

DeLong pairwise comparison of AUC values showed that the top four models (RF, CatBoost, XGBoost, and LightGBM) had significantly higher AUC values than the other six models (all *p* < 0.001). The AUC values were comparable between RF and CatBoost (*p* = 0.527) and between XGBoost and LightGBM (*p* = 0.891) but were significantly higher in RF and CatBoost than in XGBoost and LightGBM (all *p* < 0.01). Logistic regression, SVM, KNN, and BP exhibited significantly lower AUC values (all *p* < 0.001), with no significant difference between SVM and logistic regression (*p* = 0.963). Key pairwise results of the top four models are presented in Table 3.

**Table 3:** Pairwise DeLong test results of AUC for the top four models (positive cases).

Model comparison	Z-value	*p*-value	95% confidence interval (AUC difference)	Statistical significance
RF vs. CatBoost	0.632	0.527	[−0.021, 0.035]	No
RF vs. XGBoost	2.874	0.004	[0.012, 0.048]	Yes
RF vs. LightGBM	2.916	0.004	[0.013, 0.049]	Yes
CatBoost vs. XGBoost	2.765	0.006	[0.010, 0.046]	Yes
CatBoost vs. LightGBM	2.807	0.005	[0.011, 0.047]	Yes
XGBoost vs. LightGBM	0.136	0.891	[−0.020, 0.024]	No

## 4 Discussion

This is the first attempt to compare the performance of common prediction models, such as logistic regression, SVM, DT, RF, XGBoost, LightGBM, AdaBoost, CatBoost, KNN, and BP, in predicting MPPS. We collected clinical examination data used for diagnosing MPPS to build prediction models. These data consist of commonly used clinical information, which can also be accessed in underdeveloped areas with limited medical resources.

It is widely accepted that more complex models yield better non-linear fitting and improved prediction performance (20). However, our results show that a complex prediction model, such as the BP neural network, does not perform ideally in predicting MPPS. Instead, relatively simple algorithms, such as ensemble learning methods, tend to better perform in predicting MPPS. This indicates that in disease prediction, we should not solely focus on model complexity, as overly complex models may lead to overfitting and reduced prediction accuracy.

The top four models based on AUC, ranked from highest to lowest, were RF, CatBoost, XGBoost, and LightGBM. The top four models based on accuracy, ranked from highest to lowest, were CatBoost, RF, XGBoost, and LightGBM. Moreover, the top four models based on of AUDC, ranked from highest to lowest, were CatBoost, LightGBM, XGBoost, and RF. The statistical comparison of model performance further verified the superiority of the CatBoost model: Although there was no significant difference in AUC between CatBoost and RF (*p* = 0.527), CatBoost showed statistically significant advantages in accuracy, F1 score, and AUDC, metrics more closely related to clinical application (*p* < 0.05). This indicates that the CatBoost model not only has high diagnostic efficiency but also shows more stable and reliable comprehensive performance in actual clinical practice, which is more in line with the needs of MPPS auxiliary diagnosis.

Although the XGBoost model showed higher accuracy in the positive cases, its accuracy in the negative group was significantly lower than that of CatBoost. For myofascial pelvic pain syndrome (MPPS), a chronic non-fatal disease with diverse clinical manifestations, maintaining a balance between false-positive and false-negative rates is crucial in clinical practice, as both types of diagnostic errors have non-negligible clinical impacts. On one hand, false positives may not only induce unnecessary psychological anxiety in patients [anxiety that can further exacerbate pelvic pain perception (21)] but also lead to overdiagnosis and overtreatment, increasing the economic burden of patients and wasting limited medical resources (22–25). On the other hand, false negatives can lead to missed diagnosis of MPPS, delaying early intervention and standardized pelvic floor muscle rehabilitation treatment. This may cause the persistence and aggravation of pelvic floor muscle tension and dysfunction, prolonging pain symptoms and reducing patients’ quality of life.

In this study, the CatBoost model was optimized to prioritize false positive rate control, a clinical trade-off based on MPPS characteristics rather than neglecting false negatives. MPPS is a non-life-threatening chronic disease, so false positives may cause more immediate psychological and economic burdens. False negatives can be corrected by follow-up and reassessment, and conservative classification helps reduce overdiagnosis, consistent with the principle of prudent diagnosis and standardized treatment for chronic pain. Notably, this model serves only as an auxiliary diagnostic tool and cannot replace clinicians’ professional judgment. In practice, predictions should be combined with clinical symptoms, medical history, and pelvic floor examination to reduce misdiagnosis. Furthermore, CatBoost had a significantly higher AUDC than XGBoost, suggesting greater clinical net benefit. Overall, the CatBoost model demonstrates superior comprehensive performance and clinical utility for MPPS auxiliary diagnosis.

One of the contributions of this study is a comprehensive comparison of 10 models. It is not limited to evaluating metrics such as accuracy, precision, recall, F1 score, and AUC but also compares DCA and AUDC, providing a more objective evaluation of the models’ clinical utility. Based on these comparison results, we can identify more suitable models for predicting MPPS.

Another contribution of this study is the construction of a web-based graphical user interface (GUI) for predicting MPPS, which can be encapsulated to enable cross-platform use. Details of the GUI are provided in Section 4. The interface is user-friendly and intuitive, requiring no programming skills or medical knowledge from users. It holds potential for deployment in grassroots healthcare settings, assisting frontline clinicians in the diagnosis of MPPS. This is a critical advantage in an era of increasingly scarce medical resources. In addition, it can serve as a self-assessment tool for the public, as no medical expertise is needed to use it (26, 27).

Nevertheless, it should be acknowledged that all our data were derived from a single center with a relatively homogeneous study population, which may limit the generalizability of the model to other regions and medical institutions. However, it is undeniable that, as the first study to systematically compare 10 machine learning algorithms for the auxiliary diagnosis of MPPS, this research establishes a standardized research framework and provides a methodological basis for subsequent multi-center validation and clinical translation. In the future, we will conduct prospective, multi-center external validation with standardized criteria nationwide to further verify and optimize the model and improve its generalizability.

## 5 Construction of a GUI for the MPPS prediction model

**Figure 8:**
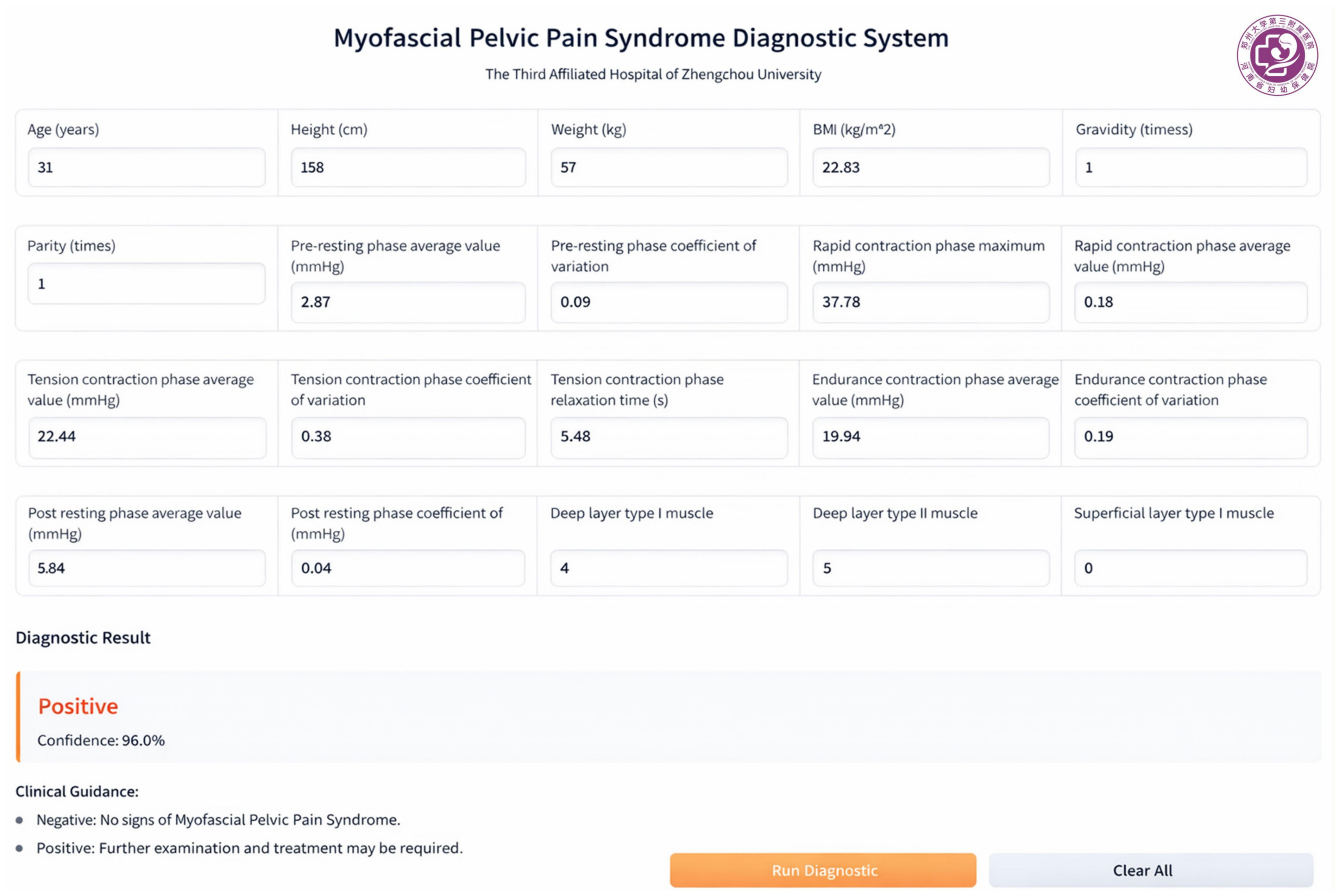
GUI for the MPPS prediction model.

The web-based GUI for the MPPS prediction model was built using Gradio and packaged for cross-platform use (Windows, macOS, and Linux), as shown in Figure 8. It consists of three modules: A modular input panel with 23 clinical features and unit annotations, a one-click diagnosis module with “Run Diagnostic” and “Clear All” buttons, and a result output module showing prediction status (Positive/Negative), confidence probability, and corresponding clinical suggestions.

Then, small-sample clinical validation of the GUI tool was performed at the Third Affiliated Hospital of Zhengzhou University from January to February 2026, including 50 female patients with suspected MPPS. Diagnoses based on the clinical gold standard were compared with those from the GUI tool. The GUI tool achieved a 92.0% diagnostic coincidence rate (46/50), supporting its good clinical applicability.

## 6 Conclusion

This study is the first to compare 10 machine learning models for MPPS prediction. Using routine clinical data, the CatBoost model showed the best performance and clinical utility for MPPS auxiliary diagnosis. A cross-platform web-based GUI tool was developed and validated, demonstrating high consistency with the clinical gold standard and good usability among medical users. This tool simplifies diagnosis, improves efficiency and accuracy, and is valuable for standardized MPPS diagnosis in primary care. Owing to the single-center design, the generalizability of the model requires further verification, and multi-center external validation will be conducted to improve the model’s reliability and clinical value.

## Data Availability

The raw data supporting the conclusions of this article will be made available by the authors, without undue reservation.
